# Clinical Profile and Outcome of Critically Ill COVID-19 Patients With Malignancy Admitted in Intensive Care Unit of a Tertiary COVID Center, India

**DOI:** 10.7759/cureus.16553

**Published:** 2021-07-22

**Authors:** Rakesh Kumar, Nishant Patel, Parul Kodan, Richa Aggarwal, Christopher Dass, Kapil Dev Soni, Anjan Trikha

**Affiliations:** 1 Department of Anaesthesiology, Pain Medicine and Critical Care, All India Institute of Medical Sciences, New Delhi, IND; 2 Department of Internal Medicine, All India Institute of Medical Sciences, New Delhi, IND; 3 Department of Critical and Intensive Care, Jai Prakash Narayan Apex Trauma Centre, All India Institute of Medical Sciences, New Delhi, IND

**Keywords:** covid, malignancy, mortality, icu, gcs

## Abstract

Introduction

There is a dearth of literature describing the clinical profile of coronavirus disease 2019 (COVID-19) in patients with malignancy. Patients with associated malignancy can have a more severe course of the disease. The aim was to study clinical course and outcome of critically ill patients admitted in ICU with associated malignancy.

Methods

The study was a single-center, retrospective, study conducted at a tertiary care hospital. Patients with active or recent malignancy on follow-up and with confirmed COVID-19 infection who were admitted to the Intensive care unit of COVID-19 dedicated hospital between November 1, 2020 to January 15, 2021 were included. Demographic data, clinical features, clinical course and outcome were retrieved from the hospital electronic medical records.

Results

A total of 24 patients with malignancy and COVID-19 were admitted to the ICU of COVID-19 center. There were 20 patients with solid organ malignancy and four patients with hematological malignancy. The most common malignancy was breast carcinoma in six (25 %) patients. Fifty percent of the patients were diagnosed with malignancy within the previous six months. Among the presenting symptoms, 13 (54.1%) patients presented with symptoms of severe acute respiratory infection (SARI), eight (33.3%) patients presented with altered sensorium, and three (12.5%) with pain abdomen. Regarding the severity of COVID-19, six (25%) patients had moderate COVID-19 and 18 (75%) had severe COVID-19. Out of 24 patients, six survived and 18 died, the mortality being 75%. The most common cause of death was sepsis with multiorgan dysfunction syndrome (MODS) in 10 (42.6 %) patients followed by severe acute respiratory distress syndrome (ARDS) and neurological cause in four (16.6 %) patients each. When survivors were compared with non-survivors, advanced age and presence of altered sensorium were more in non-survivors.

Conclusion

Severe COVID-19 and advanced malignancy is a sinister combination that has high mortality. These patients require close monitoring and aggressive care. Presence of altered sensorium and advanced age predicts poorer outcome.

## Introduction

Coronavirus disease 2019 (COVID-19) pandemic has dominated the global healthcare priorities since its outbreak early in the month of December 2019 [1]. The causative organism is severe acute respiratory syndrome coronavirus 2 (SARS-CoV-2), which is an enveloped, single-stranded RNA novel coronavirus of β-coronaviridae family [2]. COVID-19 presents with a wide variety of clinical presentation [2,3] and affects all cadre of population. There is a felt need to understand the clinical profile of this new emerging disease from across the globe, especially in immunosuppressed population who can have a more severe course of disease. It is important to focus on ‘at risk’ population and especially those with the severe spectrum of the disease. Individuals with malignancy, particularly those who are receiving systemic anticancer treatments, have been postulated to be at increased risk of mortality from COVID-19 [3,4]. Malignancy and COVID-19 is surrounded by various unanswered questions and our study is an addition to the sparse literature on COVID-19 and malignancy. Studies on COVID-19 in patients with malignancy are there [4-8] but very few related to only critically ill patients of COVID-19 admitted to ICU. We aimed to describe the clinical and demographic characteristics and COVID-19 outcomes in patients with malignancy admitted in an ICU of a tertiary care center in India. Our study is one of the first study to compile series of initial experience with severe COVID-19 and advanced malignancy from an intensive care unit at a tertiary care center from India.

## Materials and methods

The study is a single-center, retrospective study which was conducted at a tertiary care hospital after approval from local Ethics Committee, which is Institute Ethics Committee, AIIMS. Patients with active or recent cancer on follow-up who were admitted to the intensive care unit of COVID-19 dedicated hospital between November 1, 2020 to January 15, 2021 and with confirmed COVID-19 infection were included. Demographic data including the patients comorbidities, presenting symptoms, clinical course, and outcome were retrieved from the hospital electronic medical records. In the presenting symptoms, severe acute respiratory infection (SARI) has been defined by the symptoms of fever, cough and breathlessness and Altered sensorium has been defined as clinical symptoms of altered mental status such as confusion, loss of alertness, disorientation, loss of memory, disturbances in perception and poor thinking or judgement, neurological deficits and /or Glasgow coma scale (GCS) score of less than 15.

All the patients were given treatment according to the institutional protocol. The patients were initially managed on oxygen by facemask and then as the oxygen requirement increased, they were managed on high flow nasal cannula (HFNC) and non-invasive ventilation (NIV) before invasive ventilation. All patients with severe COVID-19 disease received steroids and remdesivir. Plasma was decided on case-to-case basis, based on day of presentation and clinical status. All patients received anticoagulation unless contraindicated. The length of stay in ICU was calculated as the days from admission to ICU till death or discharge from ICU .

The study has been approved by our Institutional Review Board and was conducted according to the Declaration of Helsinki.

Statistical analysis

Descriptive analysis was done by tabulation of data after testing it for normality of distribution. Qualitative variables were expressed in counts and percentages whereas quantitative variables were précised in mean with standard deviation (SD) for normally distributed data and median with interquartile range (25-75 IQR) for non-normally distributed data. No imputation was made for missing data. Univariate analysis was done using chi-square test for categorical variables and t-test (parametric) or Mann-Whitney test (non-parametric) for continuous variables.

## Results

A total of 24 patients with malignancy and COVID 19 were admitted to the ICU of COVID-19 center. There were nine males and 15 females and the median age was 44.5 years (IQR 35.5-60). The youngest patient was 14 years old and oldest 85 years old. The demographic details and co- morbidities are tabulated in Table 1. Out of 24 patients, 10 patients had comorbidities, the most common of which were diabetes mellitus, hypertension and coronary artery disease. The demographic details and co-morbidities are tabulated in Table 1.

**Table 1:** Clinical characteristics of study patients (n = 24). IQR: interquartile range; CAD: coronary artery disease; CKD: chronic kidney disease.

Characteristic	Patients median (IQR)/No. of patients (%)
Age Median (IQR) years	44.5 (35.5-60)
Male/female	9/15
Type of malignancy	
Solid organ	20 ( 83.3)
Hematological	4 (16.67)
Malignancy	
Breast	6 (25.00 )
Oral cavity	2 (8.33)
Cervix	2 (8.33)
Leukaemia	3 (12.5)
Time of diagnosis	
Newly diagnosed (during admission)	3 (12.50)
Recent within 6 months	12 (50.00)
6 month-1 year	2 (8.33)
More than 1year	7 (29.17)
Comorbidities	
No comorbidity	14 (60.87)
Diabetes Mellitus	7 (29.16)
Hypertension	3 (12.5)
CAD	3 (12.5)
CKD	1 (4.16)
1 comorbidity	6 (26.09)
>1 comorbidity	3 (13.04)

There were 20 patients of solid organ malignancy and four patients of hematological malignancy, and the most common malignancy was of breast carcinoma in six (25%) patients followed by leukemia in three (12.5%) patients. The frequency of malignancy is depicted in Table 1. Other malignancies included carcinoma lung, carcinoma gall bladder, carcinoma kidney, periampullary carcinoma, nasopharyngeal carcinoma, bladder carcinoma, stomach carcinoma, meningioma and osteosarcoma. Time of diagnosis of malignancy varied among patients from newly diagnosed malignancy to >one year of diagnosis. Fifty percent of the patients were diagnosed within the previous six months.

Among the presenting symptoms, 13 (54.1) patients presented with symptoms of severe acute respiratory infection (SARI), eight patients presented with altered sensorium, and rest three with pain abdomen. Regarding severity of COVID-19, six patients had moderate COVID-19 and 18 had severe COVID-19. The initial requirement of respiratory support varied among patients and is tabulated in Table 2. Eight patients required intubation within the first 24 hours of admission. The initial median sequential organ failure assessment (SOFA) score was 4 (3-6) in our cohort. The level of inflammatory markers and lab parameters varied among the patients and has been tabulated in Table 2.

**Table 2:** Distribution of presenting symptoms, initial clinical condition and initial inflammatory markers and duration of ICU stay in study patients (n = 24). SARI: severe acute respiratory infection; HFNC: high flow nasal cannula; SOFA: sequential organ failure assessment; IL-6: interleukein-6; GCS: Glasgow coma scale score.

Presenting symptoms	No. (%)
SARI	13 (54.17)
Altered sensorium	8 (33.33)
Abdominal pain	3 (12.50)
Initial clinical condition of the patient	No. of patients (%)/median (IQR)
Initial respiratory support	
Room air	2 ( 8.33)
Oxygen by face mask	7 (29.17)
Oxygen by HFNC	7 (29.17)
Invasive ventilation	8 (33.33)
Initial SOFA	4 (3-6)
Initial GCS	14 (13-15)
Initial inflammatory markers	Median (IQR)
IL-6 (pg/ml)	62.1 (9.1-181)
Ferritin (µg/L)	1365.5 (142-1501)
D-dimer (µg/ml)	2.86 (1.0-4.3)
Procalcitonin (ng/ml)	0.9 (0.11-2.69)
Duration of ICU stay (days)	10 (6–21.5 )

The patients were managed according to the hospital protocol. The length of stay in the ICU has been reported. The median duration of ICU stay was 10 days with IQR 6-21.5 days. Out of 24 patients, six survived and 18 died, the mortality being 75%. The most common cause of death was sepsis with MODS in 10 (42.6 %) patients followed by severe ARDS and neurological cause in four (16.6 %) patients each. The survivors were compared with non-survivors and the differences in their demographic characteristics, presenting features, inflammatory markers and outcome are summarized in Table 3.

**Table 3:** Differences between survivors and non-survivors. M:F: male:female; SARI: severe acute respiratory infection; HFNC: high flow nasal cannula; SOFA: sequential organ failure assessment; IL-6: interleukin-6; GCS: Glasgow coma scale score.

Variables	Survivors (n = 6) median (IQR)/No. of patients (%)	Non survivors (n = 18) median [IQR]/No. of patients (%)	P-value
Age (years)	35.5 (14-44)	48.5(36-62)	0.04*
M:F	1:5	10:8	0.35
Type of malignancy - Solid/hematological	4/2	16/2	0.25
Recent chemotherapy within six months	3 (50.0)	9 (50.0)	1.00
Presenting symptoms			0.03*
SARI	6 (100.0)	7 (29.1)	
Altered sensorium	0 (0.0)	8 (44.4)	
Pain abdomen	0 (0.0)	3 (16.6)	
Initial SOFA	2.5 (2-7)	5 (4-6)	0.27
GCS	15 (15-15)	13 (12-15)	0.02^*^
Initial inflammatory markers			
IL-6 (pg/ml)	25.1 ( 2.7-41.2)	87.8 ( 23.9-270)	0.14
Ferritin(µg/L)	866 (55.9-1489)	1420 (355-1501)	0.27
D-dimer (µg/ml)	2.7 (2.4-3.0)	2.8 (0.7-7.2)	1.0
Procalcitonin(ng/ml)	0.57 (0.11-19.3)	0.9 (0.11-2.6)	0.93
Duration of ICU stay (days)	14.5 (10-20)	9.5 (5-23)	0.3

## Discussion

The clinical characteristics of 24 cancer patients with laboratory-confirmed COVID-19 from COVID-19 ICU from a tertiary care center are described.

The patients with malignancy were young in our cohort with mean age being 44.5 years, and there was female preponderance. The co-morbidities were not very common in our cohort with approximately 60% of patients having no comorbidities. Other studies have found an association between co-morbidities and outcome [4,7,9]. However, our sample with co-morbidities was small to draw any conclusions.

 In our series, most (83.3%) of the patients had solid organ malignancy with breast being most common, followed by cervix and oral cavity malignancy. Among these, 40% of patients had evidence of advanced malignancy with documented metastasis and two more patients were suspected to have metastasis but further investigation to confirm the metastasis could not be done in view of critical condition at admission.

More than 60% of all cases were newly or recently diagnosed within the last six months. There was history of recent chemotherapy (i.e., within six months) in half of them and similar percentage of patients had undergone surgical treatment. However, recent anticancer therapies did not have a significant effect on mortality in our series similar to other studies [4,5,7]. 

Out of four patients with hematological malignancy, three of them were newly diagnosed to have malignancy during the present admission despite symptoms of over two months in all of them. Lockdown restrictions and a tendency to avoid a visit to the hospital facility could be a possible reason for delayed diagnosis in these patients.

Clinical presentation was typical SARI symptoms with fever and breathlessness in 54% of the patients and over one-third, i.e., eight patients presented with altered sensorium. Two of them were found to have brain metastasis, one had intracranial bleed and one had suspected brain metastasis whose imaging could not be done. One patient presented with extensive venous thrombosis; whether it had any association with prothrombotic status related to COVID-19 infection remains speculative. Cerebral venous sinus thrombosis associated with SARS-CoV-2 is a well-known complication in patients presenting with neurological complications [10,11]. Underlying malignancy could be a compounding risk factor for thrombosis. Altered sensorium was attributed to septic encephalopathy and dyselectrolytemia in other three patients.

All patients with severe COVID-19 disease received steroids and remdesivir. The need for steroids in such immunocompromised cases makes the situation more complex and challenging. The role of steroids in severe cases and the possible risk of infection was closely balanced. Even the recent guidelines have cautioned that deliberations to avoid potentially immunosuppressive therapies in oncology patients need to be precisely balanced against the overarching goal of providing optimal antineoplastic treatment. This poses a unique challenge to treating physicians [12]. In our series, the dose of steroid was closely titrated based on symptoms and immunological parameters and early tapering was done wherever plausible.

As much as 80% of the patients developed severe events in the form of ventilation or shock, and 75 % of the patients died. This series is a snapshot of clinical profile of these severe cases. Previous studies have shown a lower percentage of cancer patients developing severe events [2-8]. The main reasons for the discrepancy can be attributed to variation in the strain affecting this cohort of patients and also variation in study populations. Mortality in patients with malignancy and COVID-19 range from 6% to 30% [7,13,14,15]. In the UK Coronavirus Cancer Monitoring Project (UKCCMP), a CFR of 30.6% was observed, where 319 of the 1,044 cancer patients with COVID-19 died with 92.5% had their death attributed directly to COVID-19 [14].

The mortality rate in our series is relatively high (75%) compared to others as only sicker patients admitted in an intensive care unit of a tertiary care referral center has been included. Thirty percent of patients required invasive ventilation on the first day of admission to ICU. The more severe disease at presentation is a known bad prognostic marker. Also, the advanced stage of malignancy predicts a worse outcome.

The series includes patients on the higher end of the vulnerability spectrum when compared to all patients with cancer as these patients were majorly on active systemic therapy or advanced disease stage or presented with severe COVID-19, which can make them prone to infections and related sequelae. Additionally, some of these patients with advanced cancers have a narrow window for treatment to control disease status. Thus, a delicate balance and vigorous critical monitoring is required for their management.

Septic shock was an important cause of death in these patients. Six patients developed MDR gram-negative sepsis and succumbed to illness with *Acinteobacter baumanii* and *Kleibsella pneumoniae* being the most common organisms. The use of steroids and immunosuppressive medicines could predispose to higher chances of infection. Previous studies have also shown higher mortality in cancer patients due to nosocomial infections [16]. MDR organisms are a matter of concern and emphasize on the need for stricter infection control practices in the immunosuppressed population. A stricter and more vigilant infection control guidelines have been instituted in our ICU to minimize the risk of hospital-acquired infections.

We explored the differences between the patients with malignancy who died and those who survived the SARS-CoV-2 infection (Table 3). There was no significant difference between the survivors and the non-survivors with respect to gender, type of malignancy, and based on inflammatory markers. In addition, no significant effect on mortality was noted for the patients who had received anticancer therapy within the past month. Higher age and altered sensorium at presentation is a predictor of mortality in our series. Our finding is similar to previous studies [5,7] which showed advanced age as a predictor of worse outcome. Altered sensorium has not been the clinical presentation in most series and thus a relation to it has not been inferred in existing data. Our series include severe cases and is the possible reason for this derivation.

Strength and limitations

Our study is a brief description of the experience of COVID-19 in patients with malignancy admitted to critical care unit in a tertiary care center from India. This is one of the few compilations of COVID-19 and malignancy experience from India and depicts the severe spectrum of disease. However, our findings are also based on several study limitations. First, the study was retrospective and based on a small sample size. Second, there was heterogeneity of malignancy type and character seen. The heterogeneity of cancer types and varying stages of the disease may obscure the rationale of our findings. Some important confounders were not able to be included in the multivariate analyses, such as tumor staging due to heterogeneity of the cases included. Due to the limitations of this study, some observations need to be interpreted with caution.

## Conclusions

Severe COVID-19 and advanced malignancy is a sinister combination and has a high mortality. Use of steroids along with recent exposure to immunosuppressive agents leads to an increased risk of secondary infections and thus aggressive care is required. Presence of altered sensorium and advanced age predicted poorer outcomes in our cohort of patients. Future studies with larger sample sizes and prospective study designs can throw more light on risk factors and clinical course in COVID-19-infected patients with malignancy. More such studies can add granularity to our understanding of malignancy and COVID-19.
